# Supplementary material for: A structural rationale for reversible vs irreversible amyloid fibril formation from a single protein
Source: Nat Commun. 2024 Sep 30;15:8448. doi: 10.1038/s41467-024-52681-z (PMC11442456; doi:10.1038/s41467-024-52681-z)
Supplement: Supplementary file 1 — Supplementary Information [file 41467_2024_52681_MOESM1_ESM.pdf]

Supporting information for

A structural rationale for reversible *vs* irreversible amyloid fibril formation from a single protein

Lukas Frey[1,#], Jiangtao Zhou[2,#*], Gea Cereghetti[3,4], Marco E. Weber[1], David Rhyner[1], Aditya Pokharna[1], Luca Wenchel[1], Harindranath Kadavath[1], Yiping Cao[5], Beat H. Meier[1], Matthias Peter[3], Jason Greenwald[1], Roland Riek[1,*], Raffaele Mezzenga[2,6,*]

1. Institute of Molecular Physical Sciences, ETH Zürich, Vladimir-Prelog-Weg 2, CH-8093 Zürich, Switzerland
2. ETH Zurich, Department of Health Sciences and Technology, 8092 Zurich, Switzerland
3. Institute of Biochemistry, Department of Biology, ETH Zurich, Zurich, Switzerland
4. University of Cambridge, Department of Chemistry, Lensfield Road, Cambridge, CB2 1EW, United Kingdom
5. Department of Food Science and Technology, School of Agriculture and Biology, Shanghai Jiao Tong University, Shanghai, 200240, China
6. ETH Zurich, Department of Materials, 8093 Zurich, Switzerland

[#] These authors contributed equally to this manuscript.

*Corresponding authors: jiangtao.zhou@hest.ethz.ch, roland.riek@phys.chem.ethz.ch, raffaele.mezzenga@hest.ethz.ch

Supplementary Figure 1. AFM images of the reversible and irreversible fibril from HEWL and human lysozyme.

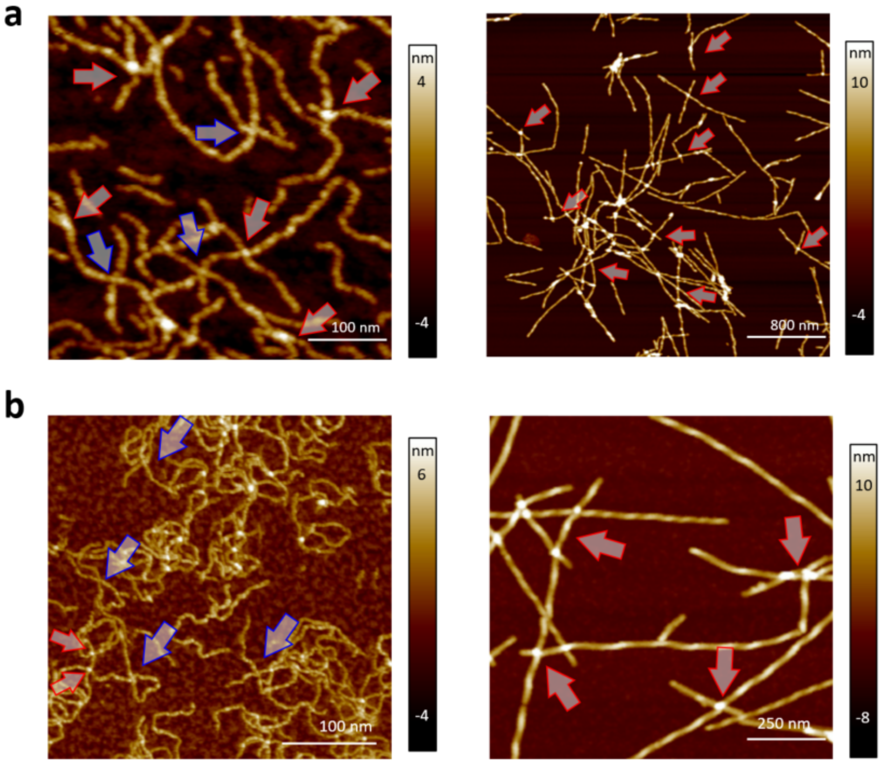

Supplementary Figure 1. AFM images showing the rigidity of the amyloid core in the reversible fibril (left) and the rigidity of the core in the irreversible fibril (right) from (a) HEWL and (b) human lysozyme. The irreversible fibrils showed an increase in their height where two fibrils overlap each other (red arrow), indicating a rigidity in their fibril core, whereas the reversible fibrils showed the fibril crossover both with (red arrow) and without (blue) a height increase, which indicates the semi-flexibility in their amyloid core.

Supplementary Figure 2. The CD spectra of reversible fibril and irreversible fibril.

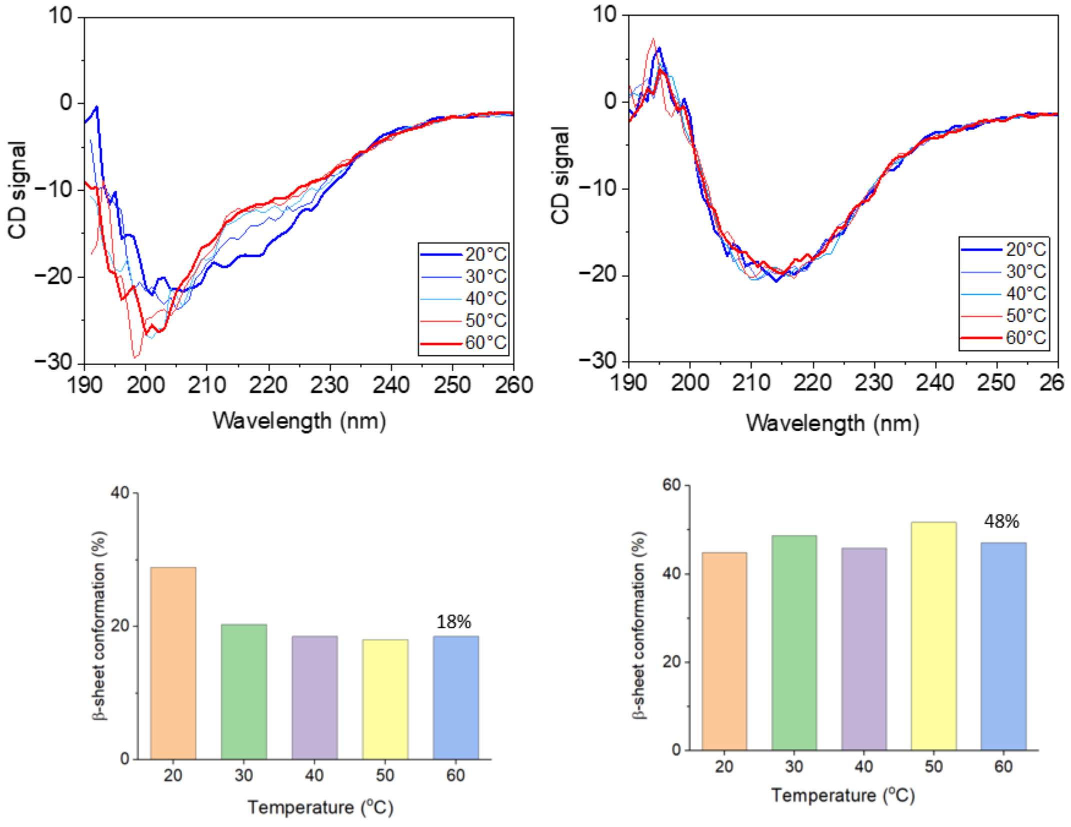

Supplementary Figure 2. The CD spectra of reversible fibril (left) and irreversible fibril (right) upon a heat treatment from 20°C to 60°C. The reversible fibril showed a structural transition around 30-40°C, while the irreversible fibril maintained their structure during the heat treatment.

Supplementary Figure 3. The CD spectrum analysis of lysozyme reversible fibril.

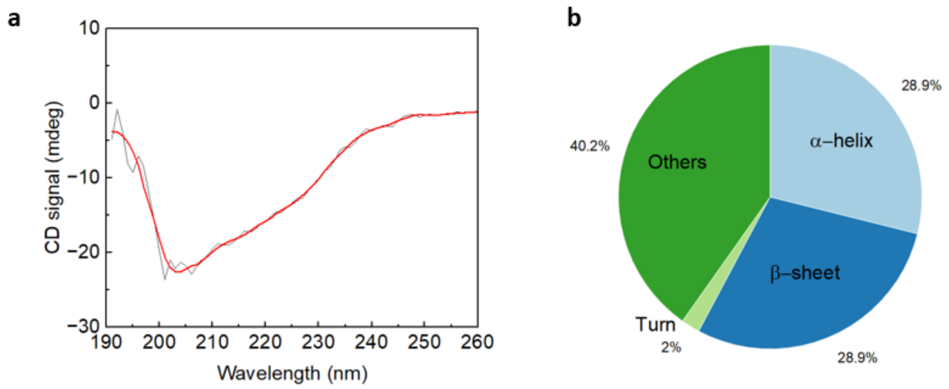

Supplementary Figure 3. The analysis of lysozyme reversible fibril CD spectrum by BeStSel algorithm shows that the reversible fibrils are composed of a mixture of α-helix, β-sheet and other secondary sctructural conformations.

Supplementary Figure 4. Cryo-EM analysis of the human lysozyme irreversible amyloid.

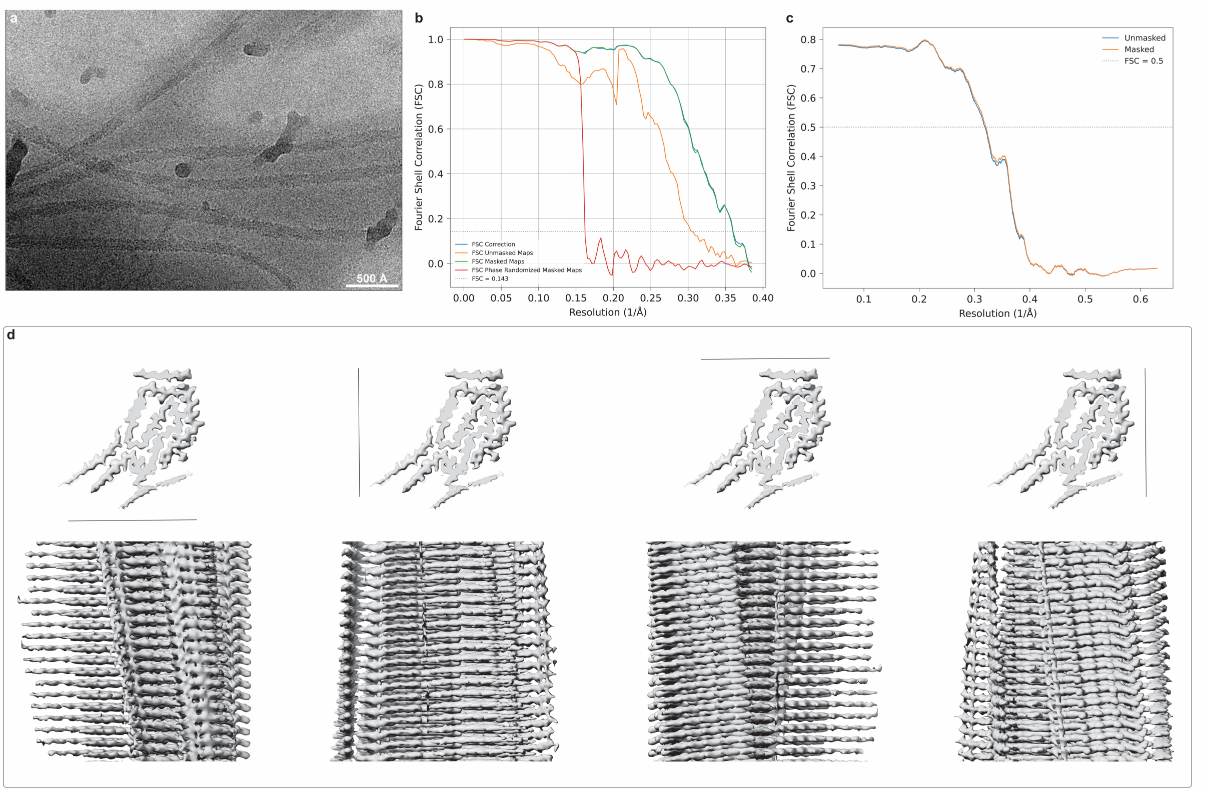

Supplementary Figure 4. Cryo-EM analysis of the human lysozyme irreversible amyloid. (a) A representative cryo-EM micrograph. (b) Half-map FSC curves for the final model. (c) Model-map FSC curves for the final 5-layer model against the map that has been cropped to within 3Å of the model. (d) Four side views of the final map showing the resolution in the direction of the helical axis. The black line near the top views of the fibril indicate the orientation of the side views below them.

Supplementary Figure 5. Cryo-EM analysis of the HEWL irreversible amyloid.

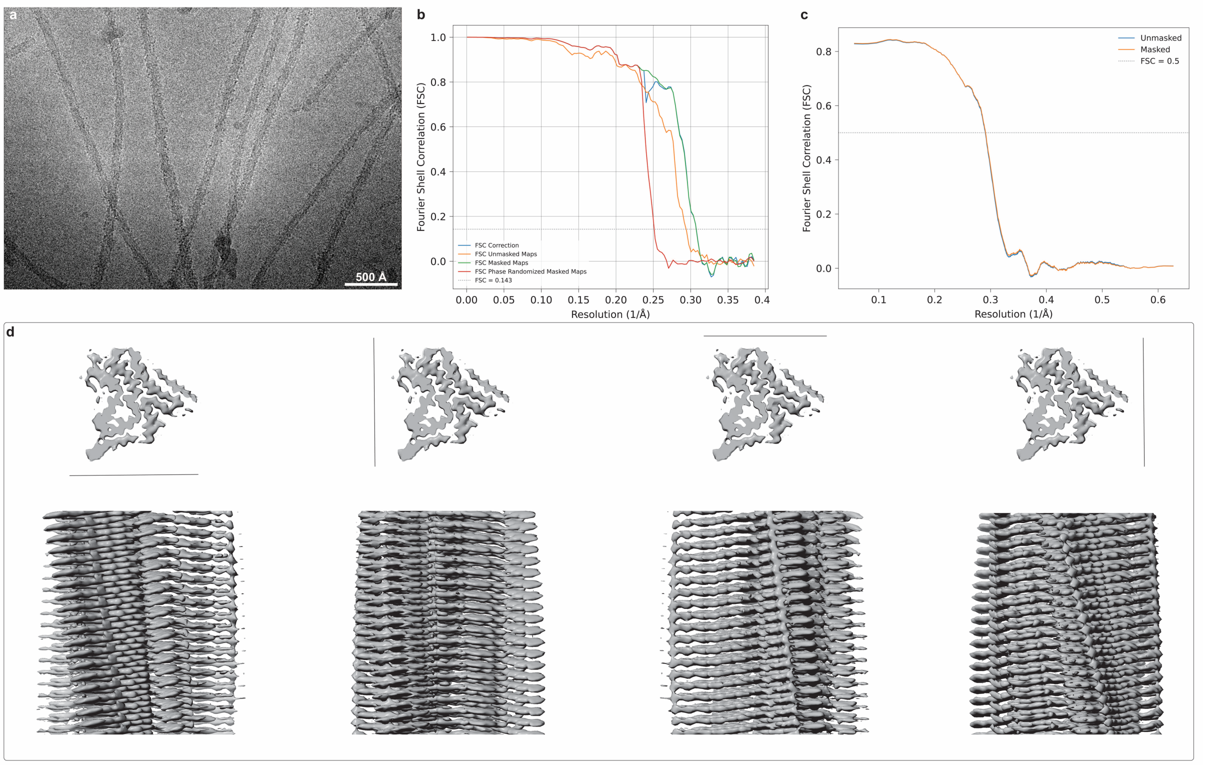

Supplementary Figure 5. Cryo-EM analysis of the HEWL irreversible amyloid. (a) A representative cryo-EM micrograph. (b) Half-map FSC curves for the final model. (c) Model-map FSC curves for the final 5-layer model against the map that has been cropped to within 3Å of the model. (d) Four side views of the final map showing the resolution in the direction of the helical axis. The black line near the top views of the fibril indicate the orientation of the side views below them.

Supplementary Figure 6. Cryo-EM analysis of the human lysozyme reversible amyloid.

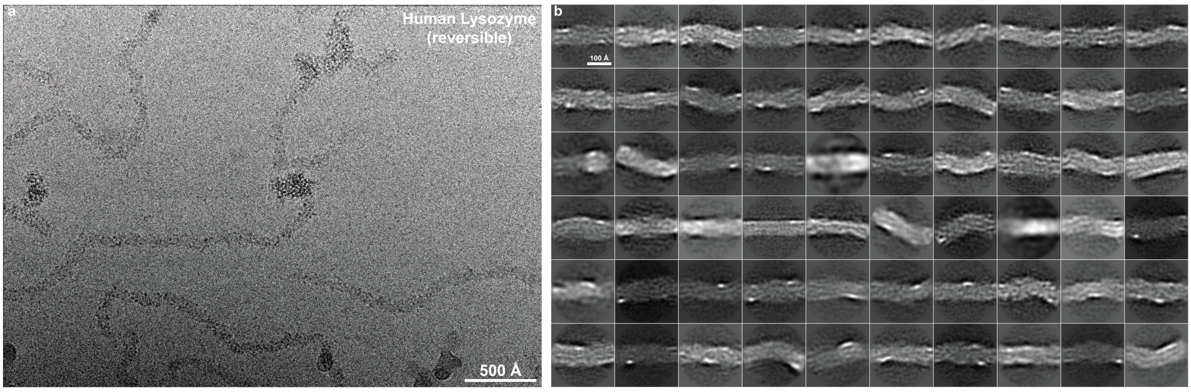

Supplementary Figure 6. Cryo-EM analysis of the human lysozyme reversible amyloid. (a) A representative cryo-EM micrograph. (b) 2D classes from a set of 66,000 particles extracted with a 260 Å box, 9.4 Å inter-box distance and a 2.6Å pixel size.

Supplementary Figure 7. 2D DARR spectrum of reversible amyloid fibrils of human lysozyme.

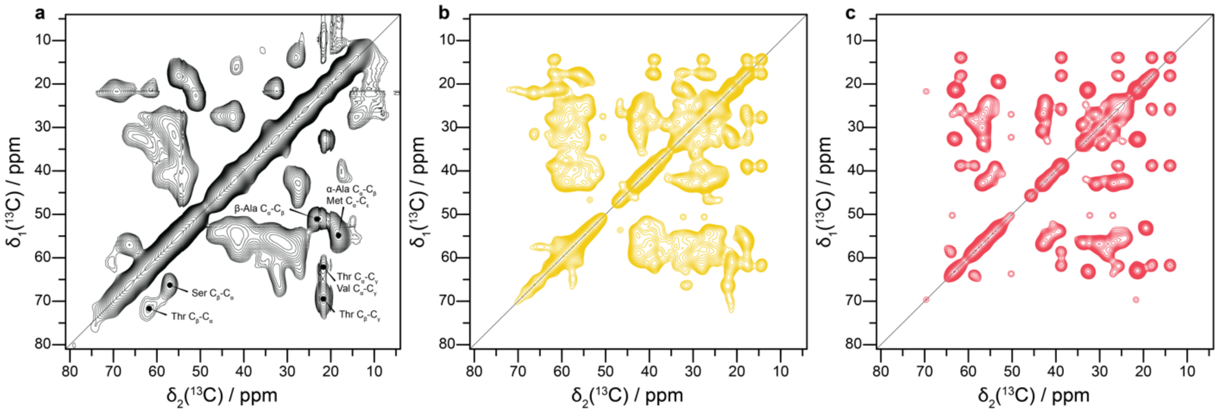

Supplementary Figure 7. 2D [^{13}C,^{13}C]- DARR spectrum of reversible amyloid fibrils of human lysozyme (a) measured on an 850 MHz ^{1}H NMR spectrometer with a few residue-specific assignments indicated (Table S3) compared to (b) the simulated spectrum (by SHIFTX) of the native human lysozyme (pdb code 7XF6) and (c) the simulated spectrum of the irreversible fibrils. A peak width of 750 Hz was applied to the simulated spectra to match the resolution of the experimental measurements.

Supplementary Figure 8. 2D NCB solid state NMR spectrum measured at 850 MHz ^{1}H frequency of human lysozyme.

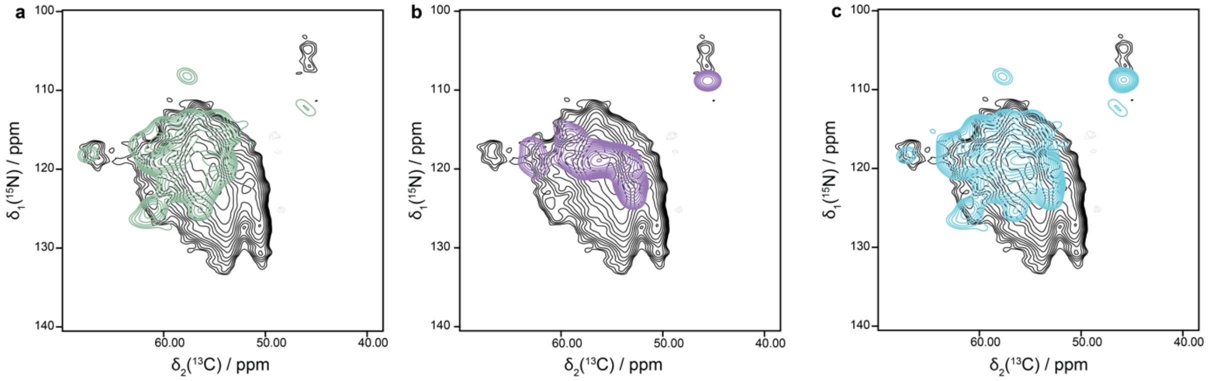

Supplementary Figure 8. Superpositions of 2D NCB solid state NMR spectrum measured at 850 MHz ^{1}H frequency (listed in Table S3) of uniformly labeled human lysozyme (black) with various simulated spectra. (a) A simulated 2D NCB spectra for the helical sub-domain A of the monomer (green). (b) A simulated 2D NCB spectra for the region of the irreversible fibril structure comprised sub-domain B residues from the native fold (purple). (c) A simulated 2D NCB spectra for a chimeric structure composed of the two structures used for (a) and (b) (cyan)

Supplementary Figure 9. MALDI-MS spectra of HEWL fibrils with different heating time.

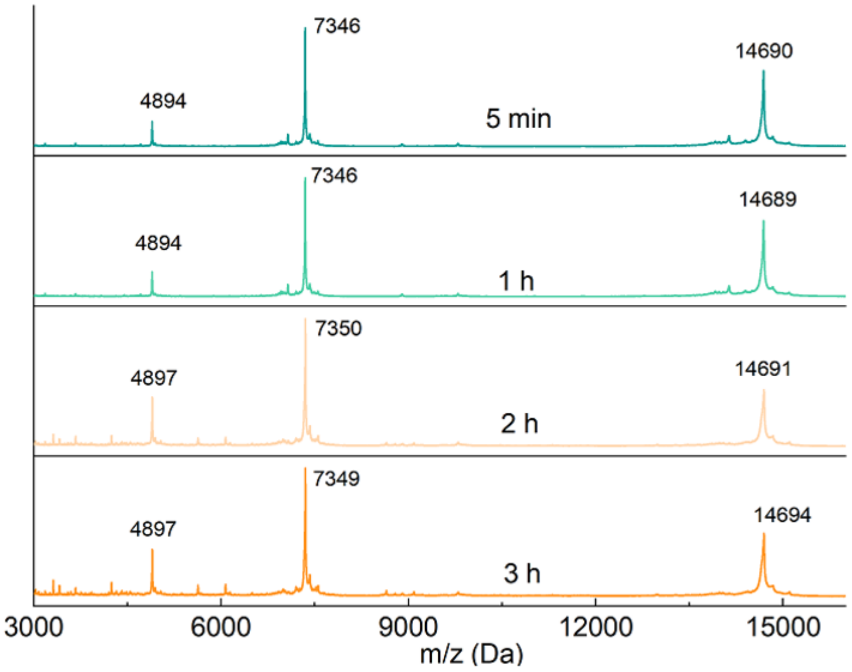

Supplementary Figure 9. MALDI-MS spectra of HEWL fibrils with different length of heating time. The peaks refers to triply, doubly and single charged ions of the full-length protein (Mw ≈ 14691 Da). These result confirms no protein hydrolysis under this incubation condition.

Supplementary Figure 10. AFM images of human lysozyme fibrils after 2.5 h incubation.

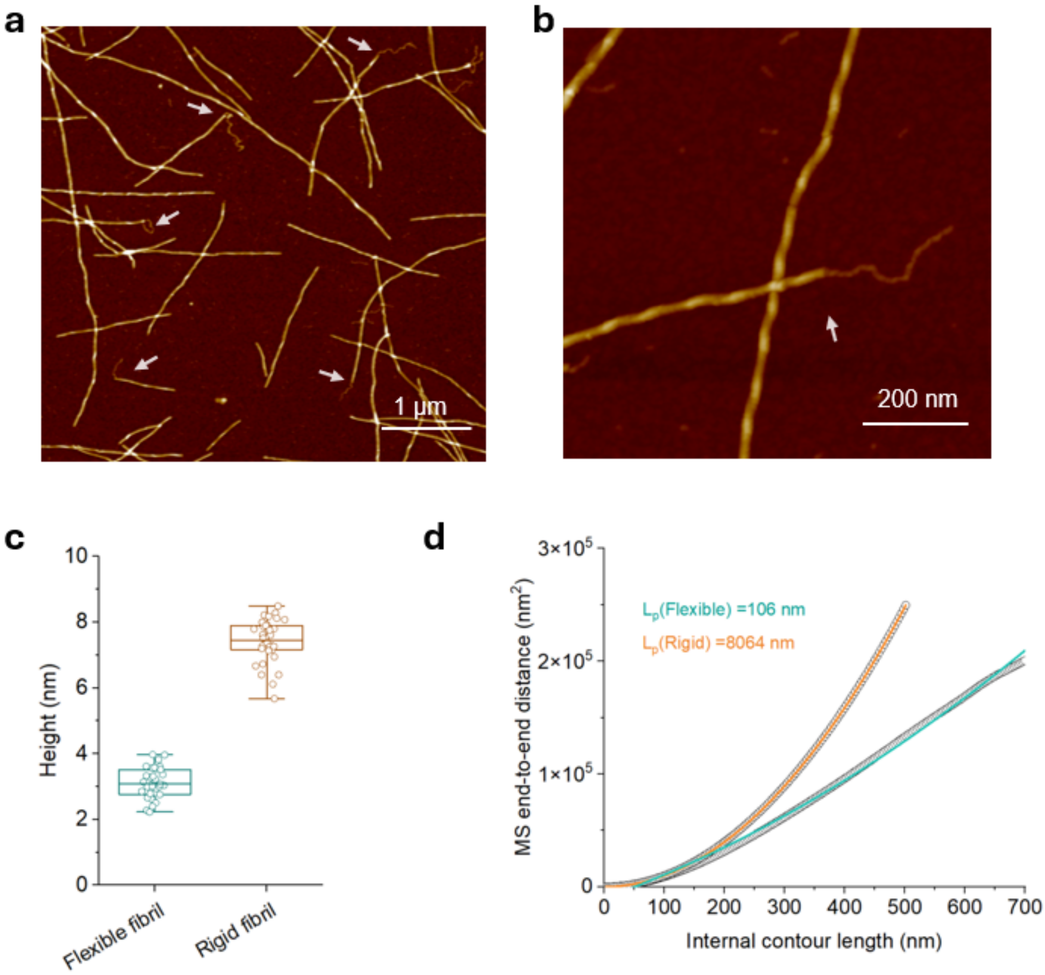

Supplementary Figure 10. The AFM images depict human lysozyme fibrils after 2.5 hours incubation. Panels (a-b) are snapshots offering insight into the transformation of remaining reversible fibrils into their irreversible counterparts. Notably, in panel b, a single reversible fibril, initially displaying high flexibility and lacking clear height periodicity, transitions into a rigid and robust irreversible fibril. Intriguingly, these conversion events manifest at both ends of the irreversible fibrils, supporting the reversible-to-irreversible fibril conversion process discussed in the main text. (**c-d**) The height and the MSED persistence length investigation of the flexible part and rigid part of the fibril showing the differences with the pure flexible and rigid fibrils from three independent experiments (n=3 independent experiments). The box plots are shown as mean value with box range of 25-75% and the min-max whiskers.

Supplementary Table 1: Line-width measurements of selected cross peaks corresponding to aliphatic moieties in the 2D ^{13}C,^{13}C DARR

δ_{F1} / ppm	δ_{F2} / ppm	Δ_{tot} / Hz
66.2	57.0	595
71.5	61.4	829
51.0	23.4	728
54.7	18.5	601
69.1	21.9	579
32.7	21.4	544
18.3	55.0	800
22.9	51.1	647
21.6	69.1	1010
27.5	42.6	1071
21.4	32.5	914
14.1	28.3	703
21.5	61.2	908
56.7	66.2	828

Supplementary Table 2: Overview on bulk ^{13}C T_2', ^{13}C $T_{1\rho}$ and ^{1}H $T_{1\rho}$ relaxation times and homogeneous linewidths for reversible lysozyme fibrils at different sample temperatures.

Temperature / °C	T_2' / ms	Δ_{homo} / Hz	^{13}C $T_{1\rho}$ / ms	^{1}H $T_{1\rho}$ / ms
-10	1.48 ± 0.04	215	4.0 ± 0.3	4.00 ± 0.23
0	1.34 ± 0.04	238	3.6 ± 0.3	3.59 ± 0.25
8	1.28 ± 0.04	249	3.3 ± 0.3	3.36 ± 0.24
18	1.17 ± 0.04	272	2.70 ± 0.24	2.99 ± 0.23

Supplementary Table 3: Overview on the protocol of flexible and rigid fibrils prepared from human lysozyme and HEWL studied in this work

Protein	Fibril	Protocol	Tests
Commerical Human lysozyme	Rigid fibril	20 mg/mL, 100 mM DTT, 10 mM NaCl, pH 7, 85 °C for 3 h, 300 rpm, ice bath for 30 min, stored at 4C	cryoEM, AFM, CD
	Flexible fibril	20 mg/mL, 20 or 100 mM DTT, 10 mM NaCl, pH 7, 85 °C for 5 min, 300 rpm, ice bath for 30 min, stored at 4C	
Purified Human lysozyme	Flexible fibril	20 mg/mL, 20 mM DTT, 10 mM NaCl, pH 7, 80 °C for 5 min, 300 rpm, RT to cool down, stored at 4C	ssNMR
Commerical HEWL	Rigid fibril	20 mg/mL, 100 mM DTT, 10 mM NaCl, pH 7, 90 °C for 5 min, 300 rpm, ice bath for 30 min, Stored at 4C	cryoEM, AFM, CD
	Flexible fibril	20 mg/mL, 20 or 100 mM DTT, 10 mM NaCl, pH 7, 90 °C for 5 min, 300 rpm, ice bath for 30 min, stored at 4C	

Supplementary Table 4: Overview on experimental parameters of the solid-state spectra measured on uniformly labeled (UL) and mixed ^{15}N-labeled and ^{13}C-labeled reversible lysozyme fibrils.

**Sample**<br>**Experiment**	Lysozyme Fibrils UL<br>DARR	Lysozyme Fibrils UL<br>NCB	Lysozyme Fibrils mixed<br>PAIN
MAS frequency / kHz	17	17	17
Field / T	20.0	20.0	20.0
**Transfer I**	**H-C CP**	**H-N CP**	**H-N CP**
^{1}H field / kHz	60	60	60
^{13}C field / kHz	34	-	-
^{15}N field / kHz	-	43	42
Shape	Tangent ^{1}H	Tangent ^{1}H	Tangent ^{1}H
Time / ms	0.9	0.7	0.6
**Transfer II**	**DARR**	**N-CB CP**	**PAIN**
^{1}H field / kHz	17	-	38.8
^{13}C field / kHz	-	6	36.7
^{15}N field / kHz	-	11	39.9
Shape	-	Tangent ^{13}C	-
^{13}C carrier / ppm	-	32.2	55.6
Time / ms	20	15	6
t_1 increments	1280	240	128
Sweep width (t_1) / kHz	50	10	8.6
Acquisition time (t_1) / ms	12.8	12.0	7.4
t_2 increments	3072	3072	3072
Sweep width (t_2) / kHz	100	100	100
Acquisition time (t_2) / ms	15.4	15.4	15.4
^{1}H decoupling / kHz	90	90	90
Interscan delay / s	2.7	2.5	2.8
Number of scans	16	512	1536
Measurement time / h	16	84	155

Supplementary Table 5: Cryo-EM structure determination and model statistics

	HEWL	Human lysozyme
**Data Collection**		
Pixel size [Å]	0.65	0.65
Defocus Range [µm]	-0.8 to -2.5	-0.8 to -2.5
Voltage [kV]	300	300
Number of frames	40	40
Total dose [e- /Å^2]	63.60	62.79
**Reconstruction**		
Reconstruction Box width [pixels]	256	256
Inter-box distance [Å]	33	33
Reconstruction Pixel size [Å]	1.3	1.3
Micrographs	822	926
Initially extracted segments	333972	168641
Segments after 2D classification	135606	85490
Segments after 3D classification	110561	51695
3D refinement Resolution [Å] (FSC > 0.143)	3.26	3.23
Final resolution [Å] (FSC > 0.143)	3.26	2.80
Estimated map sharpening B-factor [Å^2]	-72.7	-48.7
Axial symmetry	C1	C1
Helical rise [Å]	4.78	4.78
Helical twist [°]	-1.22	-1.00
**Model composition and validation**		
Non-hydrogen atoms (5 layers)	3160	3775
Protein residues (5 layers)	445	460
R.m.s. devations bond length [Å]	0.005	0.004
R.m.s. devations bond angles [°]	0.665	0.819
MolProbity score	1.69	1.86
Clashcore	5.17	3.23
Rotamer outliers [%]	0.32	1.43
Ramachandran plot favored [%]	93.83	86.82
Ramachandran plot allowed [%]	6.17	13.18
Ramachandran plot disallowed [%]	0	0
Model Resolution [Å]<br>FSC threshold	3.2 / 3.4<br>0.143 / 0.5	2.7 / 3.1<br>0.143 / 0.5
PDB code	8QV8	8QUT
EMDB-ID	18669	18663
